# Evaluation of hydroponic systems for organic lettuce production in controlled environment

**DOI:** 10.3389/fpls.2024.1401089

**Published:** 2024-08-06

**Authors:** Milon Chowdhury, Uttara C. Samarakoon, James E. Altland

**Affiliations:** ^1^ Agricultural Technical Institute, The Ohio State University, Wooster, OH, United States; ^2^ Application Technology Research Unit, United States Department of Agriculture (USDA) Agricultural Research Service, Wooster, OH, United States

**Keywords:** lettuce, liquid organic fertilizer, nutrient film technique, deep water culture, drip irrigation, Dutch-bucket

## Abstract

Organic farming methods, including the use of organic substrates, fertilizers, pesticides, and biological control, are gaining popularity in controlled environment agriculture (CEA) due to economic benefits and environmental sustainability. However, despite several studies focusing on the preparation and evaluation of liquid organic fertilizers, none have explored the compatibility of these fertilizers with different hydroponic systems. Therefore, the objective of this study was to evaluate lettuce production using a liquid organic fertilizer under different hydroponic systems. Four distinct hydroponic methods were selected: nutrient film technique (NFT), deep water culture (DWC) (liquid culture systems), and Dutch bucket (DB), regular plastic container (RPC) (substrate-based systems). ‘Green Butter’ lettuce was grown using a liquid organic fertilizer (Espartan) for four weeks. Shoot growth parameters (e.g., shoot width, number of leaves, leaf area, foliar chlorophyll content, fresh weight, and dry weight) and root growth parameters (e.g., root length, fresh weight, and dry weight) were measured. The growth difference of lettuce under the DB and RPC systems was negligible, but the growth in RPC was 29% to 60% and 15% to 44% higher than the NFT and DWC systems, respectively, for shoot width, number of leaves, leaf area, shoot fresh weight and dry weight. Root parameters were nearly identical for the NFT and DWC systems but significantly lower (21% to 94%) than the substrate-based DB and RPC systems. Although lettuce grown in the NFT system showed the least growth, its mineral content in the leaf tissue was comparable or sometimes higher than that of substrate-based hydroponic systems. In conclusion, the tested liquid organic fertilizer is suitable for substrate-based hydroponic systems; however, further evaluation of different liquid organic fertilizers, and crop species is required.

## 1 Introduction

The arable cropland is gradually decreasing throughout the world (Lambin, 2012), and soil productivity is depleting due to continuous cultivation over the years, soil pollution, acidification, loss of soil biodiversity, and increasing salinity (Blum, 1997; Lal, 2015; Gomiero, 2016). Moreover, extreme climate conditions, such as heat or cold waves, and storms, has significant impacts on crop production (Parry, 2019). As a result, an alternative crop production system needs to be adapted to ensure increasing food demand. Controlled environment agriculture (CEA) is a modern crop production approach, which can grow high quality and quantity of crops throughout the year by manipulating the ambient environmental parameters despite local geological and climatic conditions (Gruda and Tanny, 2014; Gruda et al., 2019). Moreover, hydroponic techniques under CEA facilities holds a significant promise to increase overall agricultural production using minimum resources, especially cultivation space and water (Jones, 2016; Chowdhury et al., 2021). Recently, the United States Department of Agriculture-National Organic Program (USDA-NOP) declared soilless cultivation methods as an organic production system from several aspects. Organic hydroponics is a crop culture method based on organic agriculture concepts that use organic substrate and organic nutrient solutions derived from plant and animal sources, along with the biological pest controls (Schmutz et al., 2014; Moncada et al., 2021).

Environmental conservation and sustainability are the main concepts of organic farming. It is gaining popularity among the consumers as excessive use of synthetic fertilizers and pesticides for crop production causing potential health damage and diseases, and growers are encouraged due to economic benefits and ecological awareness (Kacira et al., 2017; Ahmed et al., 2021; Murakami et al., 2021). Consumer demand for organically produced crops is increasing worldwide. According to the Nutrition Business Journal, 2022, U.S. sales of organic food products have increased by $25.1 billion from 2010 to 2021 (ERS, 2023). Since organic food began selling items in stores, sales of fresh vegetables and fruits have dominated over other types of organic food. In 2021, the annual sale of organic lettuce (*Lactuca sativa* L.) was about $276 million (NASS, 2021), as lettuce plays an important role in American diet and nutrition (Mou, 2008, 2009; Kim et al., 2016). Generally, CEA growers produce crops using water-soluble synthetic fertilizer and inorganic media (i.e., rockwool or foam).

Most organic growers typically rely on organic fertilizers such as compost manure, green manure, and bone meal for conventional (soil-based) organic crop production (Hartz and Johnstone, 2006; Gaskell and Smith, 2007). However, there are several limitations associated with using solid-state organic fertilizers in soilless cultivation systems. Nowadays, various liquid organic fertilizers are commercially available, such as Pre-Empt, Espartan, Bombardier, Caos, Tundamix, Rhyzo (Hort Americas, Fort Worth, TX, USA), Tomato & Veg Formula (Neptune’s Harvest, Gloucester, MA, USA), Grow Big (FoxFarm, Pendleton, SC, USA), and Aqua Power (SaferGro, Ventura, CA, USA). These fertilizers derive from natural plant extracts, fish emulsion, seaweed, molasses, yucca extract, humic acids, earthworm castings, kelp, fermented sugar beet molasses, wheat barley, corn, animal bone, and blood. In our previous study, three organic fertilizers were applied to lettuce alongside a conventional fertility source standardized to 100 mg·L^-1^ N to assess the efficacy of each fertilizer type. A fulvic acid-based liquid organic fertilizer (Espartan) outperformed other organic fertility sources (i.e., food scrap, yard trimming, and home compost, dairy manure). The study concluded that when standardized to the same rate of N, liquid organic fertilizer may not support yields equal to synthetic fertilizer (Floom, 2022).

Due to easy handling and availability of irrigation equipment, CEA growers prefer to use liquid organic fertilizer instead of substrate incorporated fertilizers. However, there are some major differences regarding the nutrient ion availability between organic and synthetic fertilizers. Generally, the nutrients of organic fertilizers are in a complex state that need to be broken down by microbes into their basic components before plants can absorb them. For example, organic nitrogen converts to ammonium, nitrite, and finally nitrate through the processes of ammonification and nitrification in the presence of oxygen and microorganisms (Gichana et al., 2018). Sometimes liquid organic fertilizers contain a sufficient amount of microbes, or additional microbial inoculants need to be added (Kacira et al., 2017). This dissimilation process and availability of nutrient ions may also vary based on the inoculated microbes and type of hydroponic system (i.e., open or recirculating) and hydroponic methods (i.e., nutrient film technique (NFT), deep water culture (DWC), aeroponics, ebb and flow, or containerized drip systems) as the root zone environment of each method differs. Growers typically prioritize the management of nutrient levels across various production systems, often facing limitations in crop yield and quality. Several studies have been conducted to assess crop quantity, quality, and production efficiency in different hydroponic systems (Pantanella et al., 2012; Walters and Currey, 2015; Lennard and Ward, 2019; Malik, 2021; Yang et al., 2022). In a study by Yang et al. (2022) a comparison was made between the production differences of ‘Butterhead’ lettuce in NFT and DWC systems. They observed a 13% to 27% greater growth in the NFT system for various shoot parameters, despite the use of synthetic fertilizer. In the substrate-based hydroponic systems, plastic containers and Dutch or Bato bucket are commonly used. Dutch bucket system has some advantages compared to the regular plastic containers, such as a Styrofoam lid to provide protection from light and debris. It also reduces algae growth and buffers the substrate from temperature changes. Moreover, nutrient solution usually leaches through the holes underneath the regular plastic containers, but a certain amount of nutrient solution is retained in the bucket or reservoir of the Dutch bucket, which provides more consistent substrate moisture than the regular plastic containers and influence the yield (Raj et al., 2023; Yang et al., 2023). Raj et al (2023) mentioned that the Dutch bucket system is typically used for high-wire crops, such as tomato, cucumber, pepper, eggplant, and other vine crops. However, he compared the production performance of lettuce between the NFT and Dutch bucket systems. In this study, both the Dutch bucket and regular plastic container were considered due to insufficient published information about yield variation under these systems.

Several studies were conducted to identify suitable liquid organic fertilizer from different plant and animal wastes and to evaluate their performance (Masarirambi et al., 2010; Treadwell et al., 2011; Phibunwatthanawong and Riddech, 2019; Zandvakili et al., 2019a; Nanik and Muslikan, 2021; Adekiya et al., 2022; Siddiqui et al., 2022). Some studies were also performed to compare plant growth using liquid organic fertilizers and traditional synthetic fertilizers (Zandvakili et al., 2019b). They also reported several limitations of liquid organic fertilizer usage in hydroponic crop production systems, such as biofilm formation in stock nutrient tanks, clogging of irrigation equipment, and quick mineralization and leaching (Burnett and Stack, 2009; Burnett et al., 2016; Shaik and Singh, 2022). Moreover, intensive research is required to determine the compatibility of liquid organic fertilizers for different hydroponic systems before its adoption in crop production. Therefore, this study was aimed to determine a compatible hydroponic system through lettuce cultivation using a liquid organic fertilizer under controlled environment conditions based on the growth performance, leaf tissue nutrients, and nutrient solution analysis.

## 2 Materials and methods

### 2.1 Experimental site and plant growing conditions

**Figure 1:**
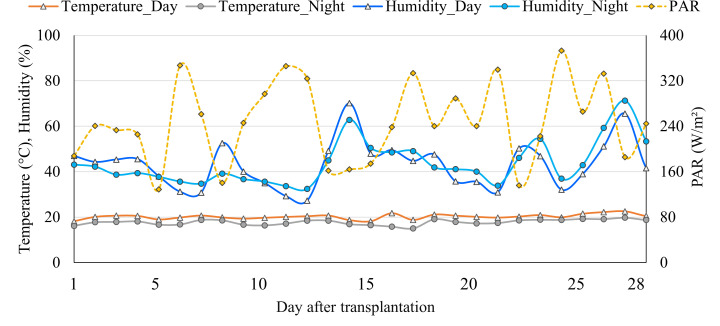
The averaged day and nighttime temperature, relative humidity, and daily averaged photosynthetically active radiation (PAR) in the greenhouse used to grow lettuce in four different hydroponic production systems.

The current study was conducted in a Venlo-type polycarbonate-covered greenhouse located at the Ohio State University, Wooster, Ohio, USA (40.7750° N and 81.9231° W), from February 24 to April 7, 2023. The greenhouse consisted of a thermal shade screen and supplemental lighting system. The ambient temperature was maintained using heater and evaporative fan-pad cooling system. The temperature set point was 20°C for daytime and 18°C for nighttime, total photoperiod was 16 hours, and supplemental lights were turned on and off when photosynthetically active radiation (PAR) was ≤ 250 and ≥350 W·m^-2^, respectively. The ambient environmental parameters and light conditions were automatically monitored and controlled by a climate control system (SEED V2, Wadsworth Control Systems, Arvada, CO, USA). However, an environmental data logger (WatchDog mini-station 2000 series, Spectrum Technologies Inc., Aurora, IL, USA) was placed at the plant height for more accurate temperature, humidity, and light intensity measurement. The averaged measured temperature, humidity (day, night), and daily average PAR per day are shown in **Figure 1**.

### 2.2 Experimental and analytical procedures

#### 2.2.1 Experimental design

**Figure 2:**
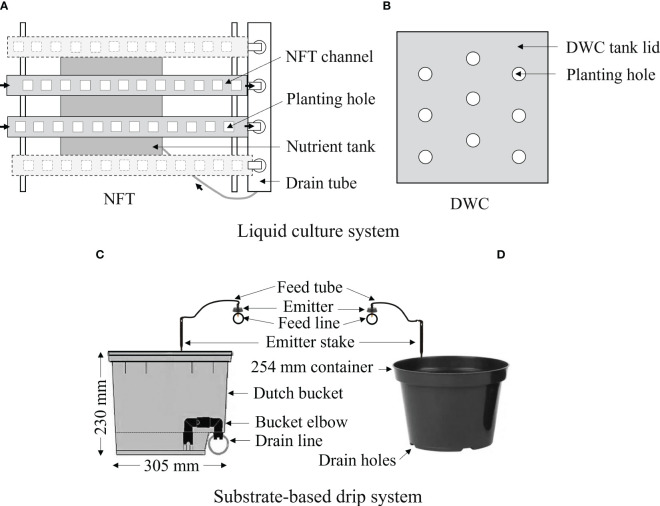
Schematic diagram of the used hydroponic systems: **(A)** nutrient film technique (NFT), **(B)** deep water culture (DWC), and **(C)** Dutch bucket (DB), and **(D)** regular plastic container (RPC) with a drip system for cultivating Green Butter lettuce using Espartan liquid organic fertilizer.

Lettuce was cultivated in liquid culture and substrate-based hydroponic cultivation systems using a liquid organic fertilizer. The nutrient film technique (NFT), deep water culture (DWC), Dutch bucket (DB) system, and regular plastic container (RPC) with drip irrigation were considered under the liquid culture and substrate-based hydroponic cultivation systems, respectively, as shown in **Figure 2**. Each NFT system was equipped with four polyvinyl chloride (PVC) channels, each measuring 122 cm in length and 13 cm in width, capable of holding 12 plants (Crop King, Lodi, OH, USA). The size of each nutrient solution tank of the NFT system was 100 liters. Only two channels from each NFT set were used in this experiment. Each DWC tank had a capacity of 100 liters and could accommodate 9 plants (Crop King, Lodi, OH, USA). A 305×254×230 mm (L×W×H) Dutch or Bato^®^ bucket and 254 mm diameter regular plastic container (Crop King, Lodi, OH, USA) were considered for the RPC and DB systems, respectively. The DB and RPC were filled with the general purpose commercial growing media (Pro-Mix-BX, Premier Tech, Rivière-du-Loup, QC, CA). Four identical sets (replicates) of NFT and DWC system (Crop King, Lodi, OH, USA), and a total of 30 RPC and 30 DB were used in this study. To have uniform dissolved oxygen (DO) conditions in solution tanks, two aeration pumps (LA-80BN, Nitto Kohki Inc., 46 Chancellor Drive, Roselle, IL, USA), one for DWC and another for NFT systems, were installed. The DO level of nutrient solution was continuously monitored using a YSI^®^ optical dissolved oxygen probe (FDO 700IQ, YSI Bioanalytical Products, Yellow Springs, OH, USA). The averaged DO level of the NFT and DWC tanks were 7.5 and 8.5 mg·L^-1^, respectively. It also helped to agitate nutrients ions. As the drip irrigation is an open-type hydroponic system and substrate helps to aerate plant roots, no aeration pump was installed in the nutrient sump tank for the drip system. Based on the lettuce growth and leachate condition, pots were irrigated with a flowrate of 150 mL·h^-1^. To prevent the evaporation of nutrient solution, each tank was covered by lid and transplanting holes without plants on the NFT and DWC systems were covered by plastic tape.

#### 2.2.2 Liquid organic fertilizer

A commercial liquid organic fertilizer (Espartan, Hort Americas, Fort Worth, TX, USA) formulated from organic matter (33.50%) and fluvic acids (19%) was used. According to the manufacturer, the fertilizer contained nitrogen at 2.70%, phosphate (P_2_O_5_) at 3.03%, and soluble potash (K_2_O) at 2.60% based on fertilizer label. The density was 1.19 g·cm^-3^ with pH from 5.8 to 5.9. A fresh nutrient solution was prepared based on manufacturers recommendation by diluting the fertilizer to a concentration of 1.58 mL·L^-1^. Then, the EC and pH (Easy Plus, Mettler-Toledo, Columbus, OH, USA), non-purgeable organic carbon (NPOC_organic carbon remaining in a sample after purging the sample with gas) and total nitrogen (TOCLCSN, Shimadzu, Kyoto, Japan), and individual nutrient ion (IC 600; Thermo Fisher Scientific, Waltham, MA, USA) of the fresh solution were measured. The EC and pH were 0.74 mS·cm^-1^, 6.84, respectively. The concentrations of the NPOC, total nitrogen, nitrite, nitrate, ammonium, phosphate, potassium, calcium, magnesium, sulfate, sodium, chloride, and fluoride were 349.50, 72.36, 0.22, 3.34, 43.78, 21.80, 44.45, 17.00, 6.49, 32.93, 47.32, 66.72, and 1.27 mg·L^-1^, respectively. As the recommended nitrogen level for hydroponic lettuce is 100-150 mg·L^-1^ (Djidonou and Leskovar, 2019), 100 mg·L^-1^ nitrogen rate was used to prepare nutrient solutions in this study. The nutrient level was maintained by maintaining EC and pH at 1.3 mS·cm^-1^ and 5.8, respectively, weekly in all systems throughout the study. As the pH level was higher than the recommended pH level (5.8 to 6.0), sulfuric acid (R8270000-10F, RICCA CHEMICAL, Arlington, TX, USA) was used to lower the pH during nutrient replenishment. The target EC (1.3 mS·cm^-1^) and pH (5.8) were checked and maintained every week throughout this study using an EC meter (COM-100, HM Digital Inc., Redondo Beach, CA, USA) and a pH meter (PH-200, HM Digital Inc., Redondo Beach, CA, USA), respectively.

#### 2.2.3 Seedling preparation, transplantation, and sampling

A commercial lettuce cultivar (*Lactuca sativa* L. var. Green Butter; Salanova lettuce, Johnny’s Selected Seeds, Winslow, ME, USA) was used for this experiment. Lettuce seeds were sown in the 2.5 cm square rockwool plugs (AO cubes, Grodan, Roermond, NL) for the NFT and DWC systems, and in plug trays filled with propagation media (Pro-Mix-BX, Premier Tech, QC, CA) for the DB and RPC systems. Before seeding, the rockwool plugs were submerged in water and allow them to soak for up to 24 hours for moisture absorption and lowering the pH. After sowing the lettuce seeds, the rockwool plugs were covered by coarse-grade vermiculite (A-3, A.M. Leonard, Piqua, OH, USA). Rockwool cubes and plug trays were kept in the greenhouse under the environmental condition as described before. The rockwool cubes and planting tray were watered regularly with a synthetic fertilizer solution (Hydro-Gro Leafy; 4.3% N-9.3% P-35% K and Ca(NO_3_)_2_, Crop King, Lodi, OH, USA) of EC and pH of 1.10 ± 0.10 mS·cm^-1^ and 5.80 ± 0.20, respectively to get uniform seedlings for transplantation. After two weeks of seeding, healthy seedlings with true leaves were transplanted into the NFT, DWC, DB, and RPC systems. The physical characteristics of the transplanted lettuce seedlings were summarized in **Table 1**.

**Table 1:** Physical characteristics of Green Butter lettuce seedlings two weeks after sowing in either a rockwool or propagation mix substrate.

Substrate	Leaf length (cm)	Leaf width (cm)	Leaf area (cm²)	Number of leaves	Relative chlorophyll^z^	Fresh weight (g)
Rock wool	3.40 ± 0.35	1.70 ± 0.10	9.49 ± 3.92	4.60 ± 0.55	36.00 ± 0.55	1.22 ± 0.05
Propagation-mix	3.82 ± 0.15	1.68 ± 0.19	10.07 ± 0.06	5.00 ± 0.00	35.90 ± 0.55	1.30 ± 0.04

^z^Relative chlorophyll content as measured by a SPAD meter.

Sample (plant) collection was performed in two steps: 14 and 28 days after the transplanting seedlings. Four replication was applied, and three lettuce plants were selected randomly and collected from each set (replication) of cultivation systems (i.e., NFT, DWC, DB, and RPC).

#### 2.2.4 Measurement of growth data

Shoot parameters, (i.e., shoot width, number of leaves, leaf area, relative foliar chlorophyll, fresh weight (FW) and dry weight (DW) of shoot) and root parameters (i.e., root length, FW and DW of root) were measured. Relative foliar chlorophyll values were measured using a commercial chlorophyll meter (SPAD-502, Minolta Corporation, Ltd., Osaka, JP). Three SPAD readings were taken randomly from fully grown lettuce leaves, and the values were averaged and recorded. Fresh weights were measured (ML3001T, Mettler Toledo, Columbus, OH), then placed in a drying oven (Heratherm OGS400, Thermo Fisher Scientific, Waltham, MA, USA) at 68°C for two days, then weighed again until a constant dry weight reached.

#### 2.2.5 Measurement of leaf tissue nutrients

Lettuce leaf nutrient analysis was conducted at Ohio State University’s Service, Testing, and Research (STAR) laboratory, Wooster, OH, USA. Total concentrations of plant essential elements including phosphorus (P), potassium (K), calcium (Ca), magnesium (Mg), sulphur (S), boron (B), iron (Fe), manganese (Mn), copper (Cu), zinc (Zn), and molybdenum (Mo) were determined by microwave digestion with HNO_3_ followed by inductively coupled plasma (ICP) (5110 ICP-OES, Agilent Technologies, Santa Clara, CA, USA) emission spectrometry according to Jones et al. (1991). Total nitrogen in plant tissue samples was determined by the Dumas method according to the Association of Official Analytical Chemists (AOAC International, 1990).

#### 2.2.6 Measurement of EC, pH, and nutrient ions of leachate and nutrient solutions

The EC, pH, organic carbon, total nitrogen, and nutrient ions (i.e., NH_4_^+^, NO_2_^-^, NO_3_^-^, PO_4_^3-^, K^+^, Ca^2+^, Mg^2+^, and SO_4_^2-^) of each replicate of the NFT and DWC systems were analyzed once a week throughout the cultivation period using the same methods and instruments mentioned as above section 2.2.2. Liquid samples were taken before the EC and pH adjustments of the NFT and DWC tanks. For analyzing the above mentioned parameters, leachate was collected from the DB and RPC systems before the final harvest (28 DAT).

### 2.3 Statistical analysis

Significance of differences between mean values were determined by analysis of variance (ANOVA) using the Minitab 21.4.2.0 software (Minitab, State College, PA, USA). Means were separated using Tukey’s multiple range test at the 5% significance level (*P* ≤ 0.05) and two-sided confidence intervals. Some basic statistical analysis was performed, and graphs were prepared using MS Excel (ver. 2023, Microsoft Corporation, Redmond, WA, USA).

## 3 Results

A significant influence of hydroponic system, on lettuce growth parameters along with the nutrient composition of edible tissue were observed.

### 3.1 Effect of cultivation systems

Hydroponic systems had significant impacts (*P* ≤ 0.05) on lettuce growth (i.e., shoot width, number of leaves, leaf area, SPAD, shoot FW and DW, root length, FW, and DW) and nutrients accumulation (i.e., N, P, K, Ca, Mg, S, B, Fe, Mn, Cu, Zn, and Mo) using liquid organic fertilizer harvested at two different times (**Table 2**). Overall, the results indicate that the efficacy of the liquid organic fertilizer largely depends on the crop cultivation methods.

**Table 2:** ANOVA test showing the effects of the cultivation system on growth variables and nutrients content of lettuce leaves at two different harvesting time.

Growth parameters	1^st^ harvest		2^nd^ harvest		Nutrient components	1^st^ harvest		2^nd^ harvest	
	F-value	P-value	F-value	P-value		F-value	P-value	F-value	P-value
Shoot width	55.65	0.000	101.20	0.000	N	3.95	0.036	99.46	0.000
No. of leaves	28.45	0.000	18.99	0.000	P	58.45	0.000	179.65	0.000
SPAD	14.31	0.000	26.91	0.000	K	17.15	0.000	90.20	0.000
Leaf area	85.98	0.000	23.06	0.000	Ca	4.35	0.027	42.48	0.000
Shoot FW	99.28	0.000	146.00	0.000	Mg	9.94	0.001	16.39	0.000
Shoot DW	57.87	0.000	72.98	0.000	S	31.56	0.000	10.75	0.001
Root length	21.02	0.000	194.44	0.000	B	25.30	0.000	23.20	0.000
Root FW	73.18	0.000	80.75	0.000	Fe	4.08	0.033	2.96	0.075
Root DW	37.63	0.000	19.22	0.000	Mn	40.93	0.000	256.90	0.000
					Cu	39.09	0.000	97.37	0.000
					Zn	39.12	0.000	26.87	0.000
					Mo	10.37	0.000	6.36	0.011

Data points are significantly different according to Tukey’s test (P ≤ 0.05), where N = 3.

### 3.2 Evaluation of growth performance

**Figure 3:**
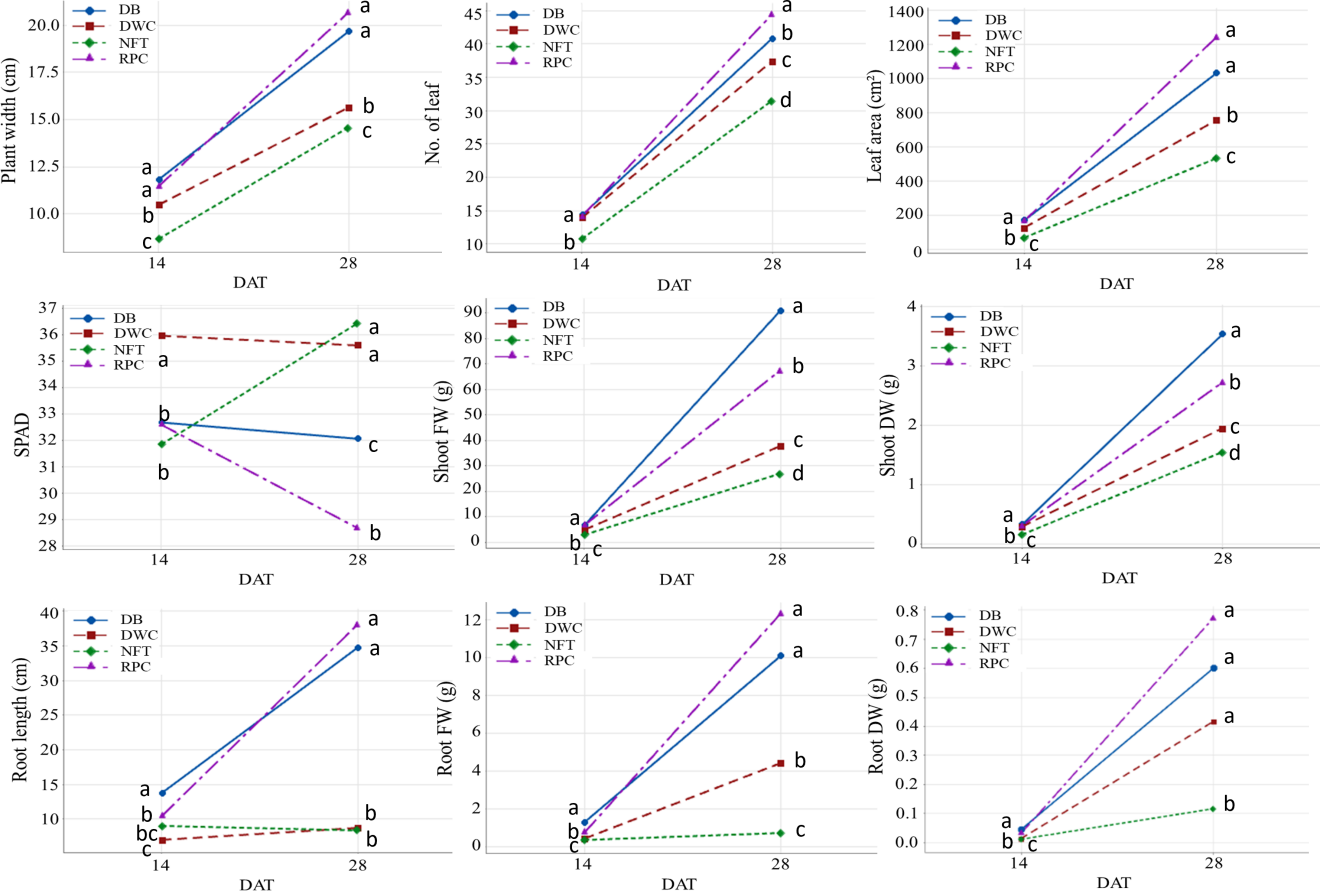
Interaction plots between nutrient film technique (NFT), deep water culture (DWC), Dutch bucket (DB), and regular plastic container (RPC) hydroponic systems and harvesting time (14 and 28 DAT) for different shoot parameters (shoot width, number of leaf, leaf area, SPAD, shoot FW and DW) and root parameters (root length, root FW and DW) of ‘Green Butter’ lettuce. Data points with different letters are significantly different according to Tukey’s test (P ≤ 0.05).

**Figure 4:**
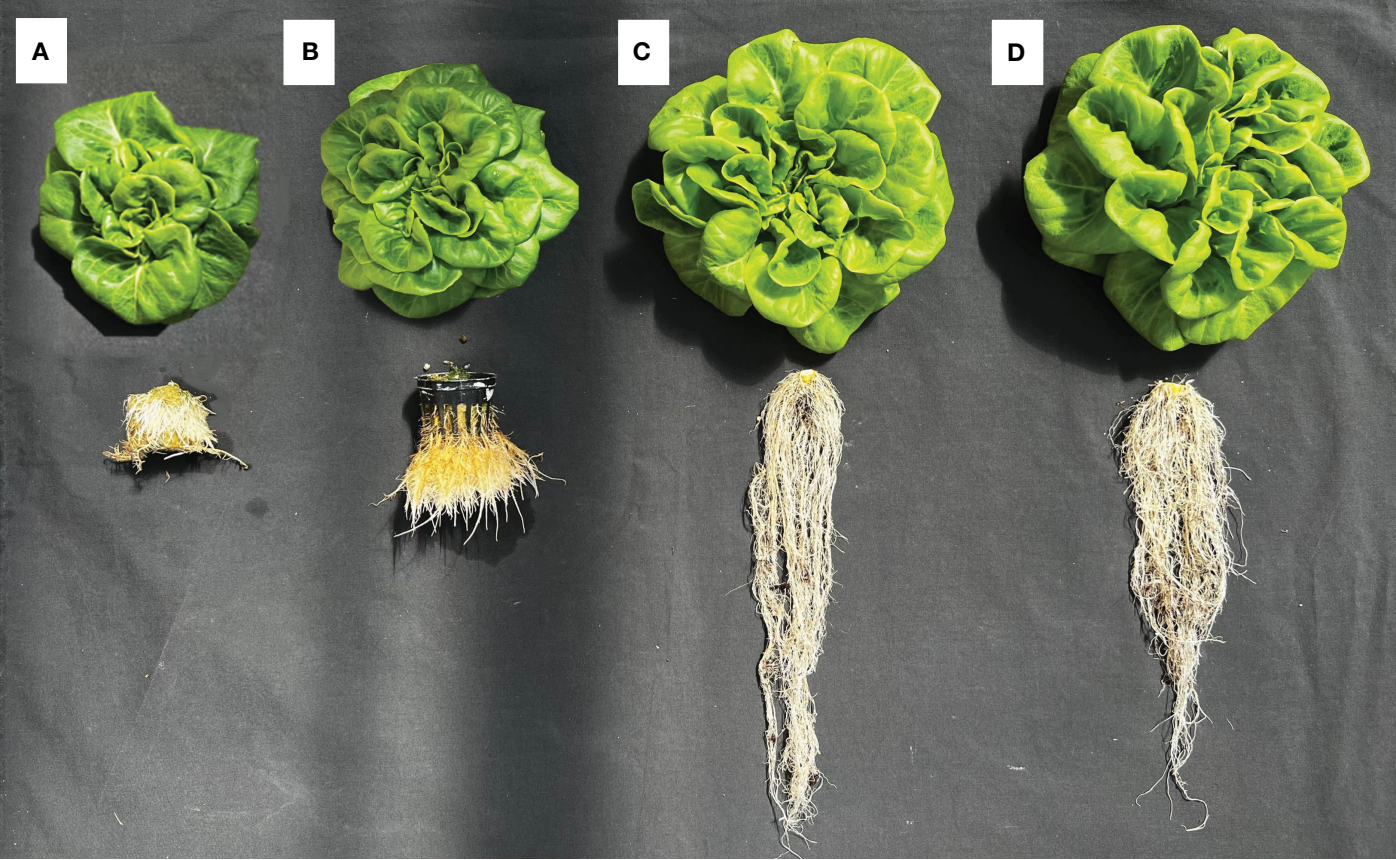
Growth comparison of representative Green Butter lettuce plants from each hydroponic system: **(A)** nutrient film technique (NFT), **(B)** deep water culture (DWC), and **(C)** Dutch bucket (DB), and **(D)** regular plastic container (RPC) harvested 28 days after transplantation.

The growth and development of lettuce between the first and second harvests for different hydroponic methods were illustrated in **Figure 3**. Plant width, number of leaves, and leaf area of lettuce grown in the RPC system were higher (ranging from 5% to 17%) than in the DB system; however, the shoot fresh and dry weight of the lettuce grown in the DB system were significantly higher (26% and 23%, respectively) than in the RPC system. Lettuce grown under the NFT system had the lowest growth performance. The root parameters (length, FW, and DW) of lettuce grown in the substrate-based systems (DB and RPC) increased in a similar pattern; however, these parameters increased less under the DWC and NFT systems. Although root length under the DWC system increased slightly with time, they were still similar to the NFT system. Lettuce grew significantly in the last 14 days; however, the growth difference pattern among the hydroponic system at the first and second harvests was almost similar, except the SPAD and root length. The percent increase in shoot FW between the first and second harvests for the NFT, DWC, DB, and RPC systems was 88%, 90%, 93%, and 91%, respectively. Although, the lettuce growth performance under the DB and RPC were similar, RPC was 42% to 151% and 19% to 79% higher than the NFT and DWC systems, respectively, for different shoot growth parameters (i.e., shoot width, number of leaves, leaf area, shoot FW and DW). However, the SPAD values of the DWC and DB systems were similar, while the NFT system showed an upward trend, and the RPC system showed a downward trend. **Figure 4** represents the shoot and root conditions of lettuce grown in the considered hydroponic systems after four weeks of transplantation. The visual observation of representative plants from the final harvest supports the quantitative data. While root DW in DWC was similar to DB and RPC (**Figure 3**), the mass of roots portrayed in **Figure 4** appears much smaller. This suggests greater root density for the DWC as compared to the DB or RPC. As the growth differences were negligible between the RPC and DB systems, the substrate-based hydroponic systems used in this trial could be considered better for liquid organic fertilizer application.

### 3.3 Evaluation of leaf tissue nutrients

Lettuce grown in the NFT system exhibited significantly higher levels of N compared to DWC at 14 DAT and DWC and RPC by 28 DAT. However, by 28 DAT foliar concentrations of P, K, Ca, B, Mn, and Cu were more abundant in the RPC and DB systems compared to the NFT and DWC systems. The DWC system demonstrated the lowest nutrients accumulation for N, P, K, and Zn by 28 DAT. Most foliar nutrient concentrations decreased over time, particularly in the NFT system. The decrease in foliar nutrient concentrations ranged from 5.7% (for Zn) to a maximum decrease of 84.7% (for Mg), except for K and Mn, which increased by 18.0% and 6.3%, respectively. On the contrary, the nutrient concentrations of lettuce grown in the DWC system generally increased, except for N (-38.19%), Ca (-5.95%), P (-24.67%), and Mg (-6.41%). The nutrient differences of lettuce grown in the RPC and DB systems were negligible, and like the NFT system, most nutrients decreased over time. There was a significant decrease in the concentration of P (22.2% to 56.2%), while K showed a significant increase (18.0% to 30.3%) across all hydroponic systems over time (**Table 3**).

**Table 3:** Summary of the leaf tissue nutrients of Green Butter lettuce grown under different hydroponic systems including: nutrient film technique (NFT), deep water culture (DWC), Dutch bucket (DB), and regular plastic container (RPC), using Espartan liquid organic fertilizer and harvested after 14 and 28 days after transplantation.

System	DAT	N (g·kg^-1^)	P (g·kg^-1^)	K (g·kg^-1^)	Ca (g·kg^-1^)	Mg (g·kg^-1^)	S (g·kg^-1^)	B (mg·kg^-1^)	Fe (mg·kg^-1^)	Mn (mg·kg^-1^)	Zn (mg·kg^-1^)	Cu (mg·kg^-1^)	Mo (mg·kg^-1^)
NFT	14	76.20 a	10.64 b	46.29 a	12.53 ab	4.07 a	4.41 a	39.90 a	337.63 a	39.85 b	105.14 a	8.54 b	0.52 a
DWC		61.10 b	7.82 c	38.62 b	10.70 b	3.39 b	3.56 c	27.20 b	268.00 ab	39.14 b	63.30 c	6.26 b	0.33 ab
RPC		71.30 ab	12.16 a	51.37 a	12.11 ab	4.06 a	4.53 a	37.60 a	217.48 b	139.35 a	100.75 ab	14.48 a	0.20 b
DB		65.70 ab	11.91 a	52.415 a	12.78 a	4.37 a	4.06 b	38.60 a	247.83 ab	180.58 a	91.45 b	14.86 a	0.21 b
Reference*		30-60	3.5-8	30-70	4.5-30	2.5-5	2.5-3.5	15-45	50-232	15-73	20-75	5-8.2	–
NFT	28	57.80 a	6.82 b	56.43 b	9.03 c	2.20 b	3.42 ab	35.30 b	236.08 a	42.55 b	99.49 a	7.27 b	0.3 ab
DWC		44.30 c	6.27 c	47.45 c	11.82 b	3.18 a	3.58 a	34.00 b	302.73 a	50.47 b	69.57 b	7.14 b	0.5 a
RPC		55.00 b	9.24 a	73.69 a	12.76 ab	3.47 a	3.19 b	37.90 a	132.18 a	220.53 a	95.71 a	12.91 a	0.2 b
DB		57.00 ab	9.75 a	74.41 a	13.15 a	3.48 a	3.20 b	39.60 a	147.35 a	230.50 a	104.04 a	14.48 a	0.2 b
Reference*		20-50	2.5-8	25-90	6-30	2.5-8	2-3.5	15-60	40-257	11-250	20-250	5-25	–

*According to Hartz et al. (2007).

Data points with different letters are significantly different according to Tukey’s test (P ≤ 0.05), where N = 3.

### 3.4 Evaluation of EC, pH, and nutrient ions of leachate and nutrient solutions

The same liquid organic fertilizer diluted to a concentration of 100 mg·L^-1^ N was supplied to the hydroponic systems (NFT, DWC, RPC, and DB) throughout the experiment. However, leachate EC, pH, and nutrient ions exhibited variations by 28 DAT (**Table 4**). The EC of the liquid culture hydroponic systems (NFT and DWC) were significantly lower (around 40%) than the substrate-based hydroponic system (RPC and DB), while the pH varied from 5.74 to 6.39. Higher ion concentrations were observed in the leachate of RPC and DB systems compared to the NFT and DWC systems. The TN and NH_4_^+^ levels were similar among all systems, but an opposite condition was observed between the NFT, DWC, and RPC, DB systems for NO_2_^-^ and NO_3_^-^ ions. A higher concentration of NO_2_^-^ ion was detected in the NFT (88.91 mg·L^-1^) and DWC (113 mg·L^-1^) systems, where a negligible concentration (1-3 mg·L^-1^) was found in the RPC and DB systems. An inverse condition was observed for the NO_3_^-^ ion. The PO_4_^3-^, Ca^2+^, Mg^2+^, and NPOC levels were significantly higher in the leachate of RPC and DB systems compared to the NFT and DWC systems, while no significant difference was observed for the K^+^ and SO_4_^2-^ ions. In general, the detected ion concentrations of the NFT and DWC (liquid culture hydroponic system) were similar, and the same scenario applied to the RPC and DB systems (substrate-based hydroponic system).

**Table 4:** Electrical conductivity (EC), pH, total nitrogen (TN), nutrient ion, and non-purgeable organic carbon (NPOC) analysis of the nutrient solution of liquid culture hydroponic (NFT, DWC) and leachate from the substrate-based hydroponic (DB, RPC) systems at 28 days after transplanting.

Parameter	NFT	DWC	RPC	DB	P-value
EC (mS·cm^-1^)	1.37 b	1.29 b	2.46 a	2.23 a	0.000
pH	7.00 bc	6.79 b	5.48 c	6.39 a	0.000
TN (mg·L^-1^)	61.25 a	64.02 a	66.83 a	64.60 a	0.913
NH_4_^+^ (mg·L^-1^)	11.50 ab	10.70 ab	8.43 c	12.34 a	0.011
NO_2_^-^ (mg·L^-1^)	17.01 b	29.73 a	0.36 c	0.86 c	0.000
NO_3_^-^ (mg·L^-1^)	0.73 b	0.73 b	116.60 a	25.10 b	0.000
PO_4_^3-^ (mg·L^-1^)	56.03 c	49.71 c	99.25 b	129.89 a	0.000
K^+^ (mg·L^-1^)	61.63 ab	71.48 ab	74.59 b	136.23 a	0.012
Ca^2+^ (mg·L^-1^)	50.70 b	48.98 b	181.05 a	146.81 a	0.000
Mg^2+^ (mg·L^-1^)	11.67 b	12.75 b	39.33 a	35.39 a	0.000
SO_4_^2-^ (mg·L^-1^)	177.13 ab	181.91 b	295.68 ab	372.38 a	0.026
NPOC (mg·L^-1^)	68.73 c	68.49 c	89.53 b	99.68 a	0.000

Data points with different letters are significantly different according to Tukey’s test (P ≤0.05), where N = 3.

## 4 Discussion

Leafy greens typically grow faster in liquid culture systems (i.e., NFT, DWC, ebb and flow, or aeroponics) as the roots receive a steady supply of essential nutrients in oxygenated water (Majid et al., 2021). However, nutrient supply might be less consistent when liquid organic fertilizer is used in a hydroponic system, as nutrients are released slowly from organic fertilizers over a period of time (Phibunwatthanawong and Riddech, 2019). The application of liquid organic fertilizer for hydroponic leafy green production is a relatively new approach, and the plant response to liquid organic fertilizer, particularly when grown in liquid culture systems, has not been fully explored. Over the last few years, most studies have compared the yield of leafy greens cultivated in substrate-based hydroponic systems using liquid organic fertilizers and synthetic fertilizers. For example, Shaik et al. (2022) evaluated six different liquid organic fertilizers for butterhead lettuce cultivation, and the NPK ratio of one of those fertilizers (AgroThrive 3-3-2) closely matched that of the fertilizer (Espartan 2.7-3-2.6) used in this study. They reported similar lettuce growth; for example, the number of leaves varied from 28 to 40 in their study, while we observed 31-45 leaves in our hydroponic systems. The leaf area ranged from 1500 to 2500 cm² in their study, whereas we found a maximum of around 1300 cm² leaf area. During leaf area measurement, we removed the curled head of the lettuce; otherwise, this parameter may have been similar as well. The fresh and dry weight of lettuce in our study varied from 30 to 90 and 1.5 to 3.5 g per plant, respectively, compared to their reported values of 80 to 150 and 8 to 12 g per plant. Variation in nutrient composition and interaction with substrate might be a reason for this weight difference. Solid matter and percentage of water in the leaves are another indications of growth evaluation. The leaf water percentage varied from 95% to 96%, mirroring the results reported by Sublett et al. (2018). Additionally, Lei and Engeseth (2021) compared the growth characteristics, functional qualities, and texture of hydroponically grown lettuce with soil-grown lettuce using synthetic fertilizer. Plant width, fresh and dry weight of hydroponically grown lettuce in their study were 21.67 cm, 45.21 g, and 6.72 g, respectively, which are similar to the results of this study. Based on the above discussion, it can be justified that the growth of lettuce was comparable to other studies with high-yield results, especially under a substrate-based hydroponic systems. Although a continuous upward growth pattern with respect of time was observed for the considered plant growth parameters with the exception of SPAD values. A downward trend over time was observed, especially among lettuce grown under the RPC system. Usually, light intensity, photoperiod, temperature, salinity, harvest time, and growth conditions affect SPAD values (Limantara et al., 2015; Xu and Mou, 2015; Cho et al., 2020). However, the selection of comparatively young leaves during the SPAD measurement at the second harvest time might be the reason for these reduced SPAD values. Theoretically, plant growth should not differ if all key environmental variables and cultivation methods are the same (Walters and Currey, 2015). In this study, the growth difference between liquid culture systems (i.e., NFT and DWC) and substrate-based drip systems (DB and RPC) was significant, but it varied closely between the NFT and DWC or DB and RPC. For example, growth variation in liquid culture systems was observed as the amount of root mass submerged in nutrient solution differs between NFT and DWC systems, leading to variations in nutrient and water uptake. In NFT, plant root tips contact a thin film of nutrient solution within plastic gutters, whereas in DWC, roots are fully submerged in nutrient solution throughout the growth process. Additionally, while NFT can provide oxygen to crops by exposing root tips to the air, an extra air pump has to be installed in DWC to maintain dissolved oxygen levels (Riggio et al., 2019; Yang et al., 2024).

Minerals in plants serve essential functions in both structural and physiological aspects. Factors influencing the growth performance of lettuce also impact its mineral composition. Despite significant variations in mineral accumulation observed due to hydroponic systems, the mineral contents were found to be similar to those reported in other research studies. For instance, Ezziddine et al. (2021) demonstrated how aquacultural sludge can serve as an organic fertilizer for hydroponic lettuce production, and the determined mineral composition aligns with the concentrations identified in this study. Similarly, Ahmed et al. (2021) reported a comparable mineral composition in lettuce grown using organic fertilizer derived from fish waste, noting that mineral content may vary with nutrient solution composition. Zandvakili et al. (2019b) observed that synthetic fertilizer contributes to high concentrations of N, K, Mg, and Fe in lettuce, while organic fertilizer leads to increased phosphorus accumulation. The macro- and micro-nutrient accumulation may vary with cultivars. Hartz et al. (2007) summarized optimal ranges of lettuce leaf minerals at different growth stages, such as early heading and preharvest, from various sources, which correspond to the first and second harvests of this study (**Table 3**).

Generally, leachate (from substrate-based culture) or nutrient solution (from liquid culture) is analyzed to determine the nutrient condition of the plant root zone (Altland, 2021; Yang et al., 2024). In organic cultivation, several studies have shown how microbial activities influence the root zoon environment and accelerate plant growth through mineralization (Niemiera and Wright, 1986; Lang and Elliott, 1997; Ruiz and Salas Sanjuan, 2022; Li et al., 2023; Wang et al., 2023). In this study, the initial EC level was 1.3 mS·cm^-1^, and the EC of the nutrient solution and leachate increased to around 1.45 and 2.4 mS·cm^-1^, respectively. The increase in EC occurred as nutrients mineralized from the organic fertilizers over time through microbial activity, which is influenced by several factors, such as growing media, temperature, moisture content, C/N ratio, oxygen supply, and pH (Niemiera and Wright, 1987; Alsanius and Jung, 2019; Sradnick and Feller, 2020; Meeboon et al., 2022). Floom (2022) explained how microbial activities are affected by growing media and cultivation time. Shinohara et al. (2011) also reported that inoculum addition is necessary to accelerate the nitrification process of liquid organic fertilizer. In this study, overall lettuce yield was significantly lower in the liquid culture systems (NFT and DWC) compared to substrate-based systems (DB and RPC), as shown in **Figure 4**. One of the reasons for this lower growth may be the improper microbial activity. Since microbes perform well in soilless substrate (Niemiera and Wright, 1986; Carlile and Wilson, 1991; Grunert et al., 2016; Floom et al., 2023) compared to liquid media, this may have led to comparatively high concentrations of nutrient ions in the leachate from substrate-based hydroponic (DB and RPC) systems. The high nitrite (NO_2_^-^) levels in the NFT and DWC systems (**Table 4**) also indicate the absence of proper inoculum or limited microbial activities, whereas the nitrate (NO_3_^-^) level was high in the DB and RPC systems, as the order of microbial oxidation of ammonia via nitrite to nitrate is sequential (Amoo and Babalola, 2017; Ayiti and Babalola, 2022). Since the sum of ammonium, nitrite, and nitrate ions was not equal to the total nitrogen, the slow nutrient mineralization or denitrification process might be responsible for this. The concentrations of P, K, Ca, and Mg ions were also higher in the DB and RPC systems due to the microbial activities. The high concentration of S ions in all hydroponic systems resulted from the addition of sulfuric acid during pH adjustment. Additionally, low root growth under the NFT and DWC systems needs further investigation as plants received high dissolved oxygen level and differences in mineral composition between systems is limited.

The pH of the plant root zone is greatly influenced by the root media (substrate) and affects nutrient availability for plant uptake (Whipker et al., 2001). For example, sphagnum peat moss and pine bark are acidic components that lower root medium pH. On the other hand, vermiculite and hardwood bark are alkaline components that raise root medium pH (Kipp et al., 2000; Sonneveld and Voogt, 2009). In this study, a lower pH was observed in the substrate-based culture (DB and RPC) systems compared to the liquid culture (NFT and DWC) systems despite weekly pH adjustments. As peat-based mixture was used as a root media in the DB and PRC systems, this reflects the pH buffering capability in substrate-based culture systems due to cation exchange at the root zone (Sonneveld and Voogt, 2009). Without the buffering ability in the root zone liquid culture systems are subject to greater fluctuations of pH in the rootzone, and therefore fluctuations in nutrient availability. Reduced levels of calcium and phosphate in the nutrient solution of NFT and DWC could also be an indication of precipitation that can occur at higher pH ranges (De Rooij et al., 1984). Therefore, in addition to nutrient content, issues related to nutrient availability may have reduced plant growth in liquid culture systems.

The application of liquid organic fertilizers in hydroponic systems holds significant future potential, driven by sustainability goals, technological advancements, market demand, and ongoing research. As research progresses, formulations of liquid organic fertilizers can be optimized to meet the specific needs of different crops, enhancing growth and yield, while also addressing current limitations, such as biofilm formation, improper nutrient mineralization, and microbial activity management. Innovations in bio-based additives and microbial inoculants could further enhance the efficacy of these fertilizers. Although, the initial cost of liquid organic fertilizers might be higher, the long-term benefits, such as improved plant health, higher yields, and premium pricing for organic produce, can make them economically viable.

## 5 Conclusions

The current study investigated the compatibility of liquid organic fertilizer with different hydroponic systems using lettuce as the model crop. The evaluation of lettuce growth performance, mineral composition, and nutrient solution analysis identified the most suitable hydroponic system for organic lettuce production. Notably, substrate-based hydroponic systems (i.e., DB and RPC) demonstrated superior performance in terms of growth parameters and mineral composition. The growth difference of lettuce between the DB and RPC systems was minimal. However, growth in the RPC system was significantly higher, with increases ranging from 29% to 60% compared to the NFT system and 15% to 44% compared to the DWC system, in terms of shoot width, number of leaves, leaf area, shoot fresh weight, and dry weight. Root parameters were nearly identical for the NFT and DWC systems but were significantly lower (21% to 94%) compared to the substrate-based DB and RPC systems. Despite the high growth and mineral composition, the substrate-based hydroponic system leached more nutrients, indicating a need for optimization. This study provides empirical evidence supporting the potential use of liquid organic fertilizer in controlled environment agriculture and the importance of selection of proper soilless cultivation methods for food production. We also suggest substrate-based hydroponic systems are a better option for screening liquid organic fertilizers for future use. In the future, it is necessary to evaluate different liquid organic fertilizers, crops, and inoculum effects to develop recommendations for organic production in controlled environment agriculture.

## Data availability statement

The raw data supporting the conclusions of this article will be made available by the authors, without undue reservation.

## Author contributions

MC: Data curation, Formal analysis, Investigation, Methodology, Software, Validation, Visualization, Writing – original draft. US: Conceptualization, Funding acquisition, Investigation, Methodology, Project administration, Resources, Supervision, Validation, Visualization, Writing – review & editing. JA: Conceptualization, Funding acquisition, Methodology, Resources, Visualization, Writing – review & editing.
